# Is there an association between inflammatory markers and lower physical performance in older adults?

**DOI:** 10.1186/s12877-022-03091-7

**Published:** 2022-05-07

**Authors:** Betty Manrique-Espinoza, Rosa Palazuelos-González, Victoria Pando-Robles, Oscar Rosas-Carrasco, Aarón Salinas-Rodríguez

**Affiliations:** 1 Center for Evaluation and Surveys Research, National Institute of Public Health, Av. Universidad #655. Colonia Santa María Ahuacatitlan, ZC 62100 Cuernavaca, Mor Mexico; 2 Center for Infectious Diseases, National Institute of Public Health, Cuernavaca, Morelos Mexico; 3 Health Department, Universidad Iberoamericana, Ciudad de México, Mexico

**Keywords:** Physical performance, Inflammation, Interleukin-10, Tumor necrosis factor-alpha, C-reactive protein

## Abstract

**Background:**

Maintenance of physical performance is essential for achievement of healthy aging. A few studies have explored the association between inflammatory markers and physical performance in older adults with inconclusive results. Our aim was to analyze the association of tumor necrosis factor-alpha (TNF-α), Interleukin-10 (IL-10), and C-reactive protein (CRP) with physical performance in a sample of older adults in rural settings of Mexico.

**Methods:**

Our study comprised 307 community-dwelling older men and women who participated in the third wave of the Rural Frailty Study. We assessed the physical performance with the Short Physical Performance Battery (SPPB) and classified older adults as low performance if SPPB scored ≤8. Inflammatory markers were ascertained using serum by immunodetection methods. Logistic regression models were used to estimate the associations between inflammatory markers and physical performance.

**Results:**

In comparison with the normal physical performance group, low physical performance individuals mainly were female (*P* <  0.01), older (P <  0.01), more illiterate (*P* = 0.02), more hypertensive (*P* < 0.01), fewer smokers (*P* = 0.02), and had higher CRP levels (P < 0.01). The logistic model results showed a significant association between the 3rd tertile of CRP and low physical performance (OR = 2.23; *P* = 0.03). IL-10 and TNF-α levels did not show a significant association.

**Conclusions:**

The results of this study were mixed, with a significant association of physical performance with higher CRP levels but nonsignificant with IL-10 and TNF-α. Further studies with improved designs are needed by incorporating a broader set of inflammatory markers.

## Introduction

The older adult population and life expectancy are growing worldwide. Given these trends, age-related decline in physical performance (PP) also increases, potentially leading to a greater number of older adults with disability [1]. Maintenance and assessment of PP are essential for physical decline prevention, independent living, and ultimately for the achievement of healthy aging. Among the instruments to evaluate PP, the Short Physical Performance Battery (SPPB) has been broadly used given its capacity to predict incident disability and all-cause mortality in older adults [2].

The physiological changes in older adults could result from the normal aging process, chronic diseases, multimorbidity, geriatric syndromes, or a combination of these conditions. In that vein, it has been shown that aging has a proinflammatory trend [3]. Although the inflammatory process is commonly a synonym for protection against trauma, injury, or infection [4], increased inflammatory markers can also lead to muscle degradation and decreased protein synthesis [5], which may contribute to impaired physical performance and functional decline.

Inflammation is a tightly regulated protective response mounted by an organism against trauma or injury [6]. The key players of inflammation are cytokines, chemokines, soluble mediators of inflammation, and acute-phase proteins (APP), among others. The duration, intensity, and variety of the stimuli factors determine the impact of inflammation on the immune response. Cytokines that have proinflammatory effects include interferon (IFN) γ, interleukin (IL) 17, IL-1β, and tumor necrosis factor-alpha (TNFα), and those with anti-inflammatory effects include IL-10, IL-4, and IL-1ra [7, 8]. However, the distinction between pro- and anti-inflammatory cytokine effects is not always clear. Also, APP, like C-reactive protein (CRP), helps to restore cytokine homeostasis [9]; and its level rises to 1000-fold during inflammatory conditions such as rheumatoid arthritis, cardiovascular disease, and infections [10].

While inflammation has been associated with morbidity and mortality in older adults [11], few studies have explored the association between inflammation and SPPB in older adults with mixed results. Sousa et al. showed that higher levels of CRP were associated with scores < 8 in SPPB in a sample of older adults from five countries [12]. Nonce, no significant associations were found in the study from Legrand et al. with older adults from Belgium [13]. Regarding levels of TNF-α, results are also inconclusive. First, no significant associations were found with SPPB scores in the studies from Cesari et al. and Brinkley et al. [14, 15]. Second, Hsu et al. identified two main combinations of inflammatory biomarkers (TNF-α related component and CRP-related component), and higher levels of TNF-α were inversely associated with lower physical performance battery score [16]. Similar results were found in Calvani’s study. Regarding anti-inflammatory cytokine IL-10, only two studies have explored its association with SPPB, one study with no significant associations reported [14], and the other reported a significant association with higher levels of IL-10 [17].

The research on inflammation and its association with health outcomes among older adults is complex given that different cytokines and APP are involved in the inflammatory cascade and their variability depending on the stage of inflammation (acute or chronic). In that vein, the inclusion of various inflammatory biomarkers could provide a higher insight into the association between inflammation and PP. Furthermore, little is known about this association in low- and middle-income countries, especially among the most disadvantaged populations. Therefore, this study aimed to analyze the association of TNF-α, IL-10, and CRP with PP among a sample of community-dwelling older adults in rural settings of Mexico.

## Methods

### Study design and participants

Data from the third wave of the Rural Frailty Study (RFS), collected in 2018, was used. The RFS is a longitudinal study with baseline wave in 2009 and two other waves (2013, 2018). The methodological details have been described elsewhere [18, 19]. Briefly, it is a prospective cohort study whose principal aim was to determine the prevalence and incidence of frailty among rural older adults in Mexico. The baseline sample size was 600 individuals [18]. We included A small refreshment sample in each follow-up measurement to compensate for mortality and follow-up losses [18, 19]. In wave 3 (2018), 566 face-to-face interviews (483 follow-up and 183 refreshing sample) were collected. From these, 88 died during waves 2 and 3, and 35 were lost at follow-up. For wave 3, venous blood samples were collected in a subsample of 361 older adults also. The Research and Ethics Committee of the National Institute of Public Health, Mexico, approved the study. The older adults signed informed consent before data collection.

### Analytical sample

For the present study, 307 men and women who had complete data for all study variables were included. Excluded participants (*n* = 54) were more female, older, had more limitations in basic and instrumental activities of daily living, lower vigorous physical activity (i.e., less time spent in vigorous physical activity), and lower tobacco and alcohol use (*P* < 0.05). No significant differences were observed in the remaining study variables (IL-10, CRP, TNF-α, diabetes, hypertension, ethnicity, and literacy).

### Physical performance

Physical performance was assessed with the Short Physical Performance Battery (SPPB), consisting of three tests: walking, standing balance, and chair stand performance. For the walking test, participants were asked to walk 4 m at the usual pace. For the chair–stand test, participants were asked to fold their arms across their chest and stand up from a sitting position and sit down five times as quickly as possible. For the balance test, participants were asked to stand in a tandem position. Each task was assigned a score from 0 (inability to complete the task) to 4 (best performance). A summary performance score was obtained by the SPPB test (0 to 12). For analyses, the participants were classified as having low physical performance if score < 8 [2].

### Inflammatory markers

Fasting blood venous samples were drawn and centrifuged in situ to obtain serum. Subsequently, the samples were stored at − 70 °C in liquid nitrogen in coded cryovials until their analysis in the National Institute of Public Health laboratory. Concentrations of IL-10 and TNF-α were measured using the Milliplex™ MAP Human Cytokine/Chemokine Magnetic Bead Panel (EMD Millipore Corporation, Billerica, MA, USA). Sensitivity of IL-10 and TNF-α were 1.1 pg/ml and 0.7 pg/ml, respectively. Intra-essay variability for IL-10 was ±1.6, and inter-essay variability of ±16.8, for TNF-α ±4.1 and 9.5, respectively. CRP was determined using a human CRP ELISA kit (Item No. CYT298; Millipore) with a 0.20 mg/mL sensitivity, intra- and inter-assay variability were respectively ±4.6 and ± 6.0%. Both methods were performed according to the manufacturer’s instructions.

### Covariates

Sociodemographic characteristics including age, sex (female = 1), ethnicity (1 = self-report about speaking an indigenous language), literacy (yes = 1), current alcohol consumption (yes = 1), and smoking habit (1 = consumed at least 100 cigarettes in their life) were considered. The presence of hypertension or diabetes was determined by self-report of a previous medical diagnosis. The limitations in basic activities of daily living (ADL) were defined as having difficulty carrying out at least one of the following tasks: bathing, dressing, toileting, transferring, continence, and feeding, using the Katz ADL Index [20]. As for limitations in instrumental activities of daily living (IADL), the following tasks were evaluated: using a telephone, doing shopping, handling medicine, managing money, using public or private transportation, and, just for women, the ability to prepare meals, do housekeeping, and do laundry. The Lawton and Brody scale was used to assess these activities [21]. Body mass index (kg/m2) was categorized as follows: underweight (< 18.5), normal weight (≥18.5–24.9 kg/m), overweight (25.0–29.9 kg/m), and obese (≥30.0–34.9 kg/m) [22]. Physical activity (PA) was measured with the short form of the International Physical Activity Questionnaire (IPAQ) that assesses specific types of activities like walking, moderate-intensity activities, and vigorous-intensity activities expressed in Metabolic Equivalent of Task (MET) mins/week. OA were classified in Low PA (those who do not meet the quantity or intensity of moderate PA), Moderate PA (5 or more days of moderate-intensity activity or walking of at least 30 minutes per day), or High PA (vigorous-intensity activity on at least 3 days achieving a minimum total physical activity of at least 1500 MET-minutes/week) according to with their IPAQ scores [23].

### Statistical analysis

The participant characteristics were described using means or proportions as appropriate. Chi-square and Mann-Whitney-Wilcoxon tests were carried out to compare PP groups regarding sociodemographic, health, and lifestyle variables. We adjusted several logistic regression models to estimate the association between CRP, IL-10, and TNF-α and low physical performance. First, a model with no covariates; second, a model adjusted for sociodemographic and lifestyle variables (age, sex, literacy, ethnicity, tobacco use, alcohol consumption); and third, a model adjusted for health variables (diabetes, hypertension, limitations in ADLs and IADLs, body mass index and physical activity). CRP, IL-10, and TNF-α values were categorized into tertiles in all models, with the first tertile (lower values) as the reference category. Differences were considered statistically significant if *p*-value < 0.05.

## Results

The following general characteristics were observed in the analytical sample. Three hundred seven older adults were included, and the prevalence of low physical performance was 57%. The mean age was 80.6 (SD = 3.6), 49.5% were female, 57.7% were illiterate, 53.7% had an ethnic background, and 43% had a partner (married or cohabited). Regarding BMI, 31.6% had overweight, 8.8% obesity, 7.2% were underweight, and 52.4% adequate weight. 14.3% reported diabetes and 49.5% hypertension. 19.9% currently consumed alcohol, 35.8% consumed at least 100 cigarettes in their life.

Table 1 shows the sociodemographic characteristics and health conditions of participants by physical performance status. In comparison with the normal physical performance group, low physical performance individuals mainly were female (*P* < 0.01), older (P < 0.01), more illiterate (*P* = 0.02), more hypertensive (P < 0.01), and fewer smokers (*P* = 0.02).

**Table 1:** Characteristics of the study participants by physical performance

Variables	Overall (***n*** = 307)	Physical Performance				***p***-value*
		Normal		Low		
		***n*** = 132	43%	***n*** = 175	57%	
	%	n	%	n	%	
Female	49.5%	49	37.1	103	58.9	< 0.01
Age (mean ± SD)**ª**	80.6 ± 3.62	132	79.8 ± 3.62	175	81.2 ± 3.50	< 0.01
Ethnicity (1 = self-report about speaking an indigenous language)	53.7%	72	54.55	93	53.14	0.81
Literacy (yes = 1)	42.3%	66	50.0	64	36.6	0.02
Smoking habit (1 = consumed at least 100 cigarettes in their life)	35.8%	57	43.2	53	30.3	0.02
Alcohol consumption (yes = 1)	19.8%	31	23.5	30	17.1	0.17
Limitation in ADL	21.8%	18	13.7	49	28.0	< 0.01
Limitation in IADL	55.1%	47	35.6	122	69.7	< 0.01
Hypertension	49.5%	53	40.2	99	56.6	< 0.01
Diabetes	14.3%	14	10.6	30	17.1	0.11
Body mass index (mean ± SD)ª	24.7 ± 4.1	132	23.9 ± 4.0	175	24.7 ± 4.1	0.09
Body mass index						
Underweight	7.2%	11	8.3	11	6.3	
Normal	52.4%	74	56.1	87	49.7	0.35
Overweight	31.6%	39	29.6	58	33.1	
Obese	8.8%	8	6.1	19	10.9	
Physical Activity						
Low	42.0%	30	22.7	99	56.6	
Moderate	32.9%	50	37.9	51	29.1	< 0.01
High	25.1%	52	39.4	25	14.3	
Physical Activity [MET – minutes/week] (mean ± SD)ª	2516.8 ± 4086	132	3955.8 ± 5158.6	175	1431.451 ± 2564.5	< 0.01
SPPB total score (0–12 poinst) ^b^	6.62 ± 2.75	132	9.05 ± 1.17	175	4.79 ± 2.09	< 0.01
SPPB - Balance score^b^	2.85 ± 1.35	132	3.78 ± 0.5	175	2.86 ± 1.35	< 0.01
SPPB - Walking score^b^	2.47 ± 1.05	132	3.25 ± 0.57	175	2.46 ± 1.05	< 0.01
SPPB- Chair/ stand score^b^	1.32 ± 1.07	132	2.02 ± 1.08	175	1.32 ± 1.07	< 0.01

*ADL* Activities of Daily Living, *IADL* Instrumental Activities of Daily Living

ªmean ± SD

^b^U de Mann Whitney

*Chi-square or t tests

Table 2 shows the comparisons of CRP, IL-10, and TNF-α levels across physical performance groups. Higher levels of CRP were observed in participants with low PP (*P* = 0.01) in contrast with the normal PP. No significant differences were observed for IL-10 (*P* = 0.12) and TNF-α (*P* = 0.29). The observed ranges for each tertile of the inflammatory markers were the following. For CRP < 1.6 mg/L, ≥1.6 mg/L thru < 5.7 mg/L, and **≥** 5.7 mg/L, for 1st, 2nd and 3rd tertile, respectively. For TNF-α values were: < 26.0 pg/ml, ≥26.0 pg/ml thru < 38.1 pg/ml, and **≥** 38.1. And for IL-10, were: < 1.63 pg/ml, ≥1.63 pg/ml thru < 6.3 pg/ml, and **≥** 6.3.

**Table 2:** Levels of inflammatory cytokines by physical performance group

Variables	Overall (***n*** = 307)		Physical Performance				***p***-value*
			Normal (***n*** = 132)		Low (***n*** = 175)		
	Median	IQR	Median	IQR	Median	IQR	
CRP (mg/L)	3.15	6.61	2.62	5.08	3.62	7.6	0.01
IL-10 (pg/ml)	3.83	6.84	3.56	5.85	4.89	7.19	0.12
TNF-α (pg/ml)	30.94	19.32	29.98	18.89	33.00	19.48	0.29

*IQR* Interquartile range

*Mann-Whitney-Wilcoxon test

Table 3 shows the results of the logistic regression models for the associations between inflammatory markers and PP. The unadjusted model showed a significant association between the 3rd tertile of CRP and low physical performance, OR = 1.83; CI95%: 1.06–3.12 (Model 1, Table 3); which persisted when sociodemographic and lifestyle covariates were controlled, OR = 2.16; CI95%: 1.20–3.90 (Model 2, Table 3). Finally, in the completely adjusted model (Model 3, Table 3), also controlled for health-related variables, significant relationships were observed between 3rd tertile of CRP with low PP (OR = 2.23; 95%CI: 1.10–4.55). No significant differences were observed for IL-10 and TNF-α.

**Table 3:** Associations of inflammatory markers with low physical performance

	Model 1		Model 2		Model 3	
	OR	(95% CI)	OR	(95% CI)	OR	(95% CI)
CRP (mg/L)						
1st Tertile (< 1.6) (reference)	1.00		1.00		1.00	
2nd Tertile (**≥**1.6 thru < 5.7)	1.37	(0.81–2.30)	1.38	(0.79–2.42)	1.81	(0.93–3.50)
3rd Tertile (**≥**5.7)	1.83	(1.06–3.12)**	2.16	(1.20–3.90)**	2.23	(1.10–4.55)**
IL-10 (pg/ml)						
1st Tertile (< 1.63) (reference)	1.00		1.00		1.00	
2nd Tertile (**≥1.63** thru < 6.3)	0.92	(0.53–1.57)	0.91	(0.52–1.63)	0.90	(0.46–1.77)
3rd Tertile (**≥6.3**)	1.26	(0.72–2.20)	1.24	(0.68–2.24)	1.12	(0.55–2.26)
TNF-α (pg/ml)						
1st Tertile (< 26.0) (reference)	1.00		1.00		1.00	
2nd Tertile (**≥26.0 thru** < 38.1)	1.01	(0.59–1.71)	1.15	(0.65–2.03	1.22	(0.63–2.39)
3rd Tertile (**≥38.1**)	1.16	(0.66–2.03)	1.19	(0.66–2.17)	1.22	(0.60–2.47)

Model 1: Logistic regression without covariates

Model 2: Additionally adjusted for sex, age, ethnicity background, literacy, alcohol consumption, and smoking habit

Model 3: Additionally adjusted for limitation in ADL, limitation in IADL, presence of hypertension, diabetes, physical activity, and body mass index

**p < 0.05

## Discussion

The results of our study show that inflammation is partially associated with low physical performance among Mexican older adults living in rural settings. Specifically, higher levels of CRP, but not IL-10 and TNF-α, are associated with lower physical performance.

Regarding the association of CRP and low physical performance, evidence from previous studies has reported mixed results. Brinkley et al. identified, in one study with more than 500 participants over 55 years, that higher values of CRP were associated with lower scores of SPPB (β = − 0.21, *P* < 0.01), in models adjusted by age, gender, race, and body composition [18]. Another study, with African American participants, reported similar results (β = − 1.426, *P* < 0.001) [24]. An additional multicultural study, comprising 1371 participants from Brazil, Colombia, Albania, and Canada, reported that OA with CRP ≥10 mg/l had lower levels of PP using the SPPB [12]. Meanwhile, other studies have not found significant associations. Legrand et al. found that CRP was not associated with SPPB in Belgian women over 80 years [13]. Hsu et al. reported physical performance battery score not related to the CRP-related component [16]. Cesari et al. used a summary score considering gait speed, chair stand test, and standing balance test to assess PP in Italian OA and found that CRP was not associated with the summary score for PP. [14]

Evidence regarding the associations of IL-10 and TNF-α with PP is scarce but most is consistent with our findings. Cesari et al. identified that IL-10 was not associated with the summary score of PP (β =0.01, *P* = 0.60). Similarly, Brinkley et al. and Cesari et al. did not observe significant associations between TNF-α and PP (β = 0.13, *P* > 0.05; and β = 0.01, *P* = 0.55, respectively). Meanwhile, other studies have found significant associations. Hsu et al. found that TNF-α-related component was associated with SPPB. Calvani et al. found that higher concentrations of IL-10 and TNF-α in older adults with low-functioning (SPPB ≤8) [17]. The lack of consistency in the reported results can be explained by different measures of physical performance and heterogeneity in the populations analyzed.

Inflammation is a complex response that is involved in different body homeostatic processes. However, the specific mechanisms from which it acts on PP have not been fully elucidated. Although it has been proposed that inflammation could negatively affect PP via a decrease in muscle mass and strength. A recent literature review from Tuttle et al. reported that higher levels of inflammatory biomarkers were associated with lower muscle mass and muscle strength in different OA populations. In special, higher levels of CRP, IL-6 and TNF-α were associated with lower handgrip and knee extension strength [25]. Calvani et al. in their exploratory study with 35 older adults reported the older adults with Low- functioning (SPPB ≤8) were characterized by lower muscle volume and knee extensor strength compared with high-functioning (SPPB> 8) participants [17].

Regarding the mixed results observed in our study, CRP, but not IL-10 and TNF-α, significantly related to low physical performance, a systematic review and meta-analysis of 17 studies with 11,249 individuals also showed that only CRP, but not TNF-α and IL-6, is associated with sarcopenia [26]. Overall, inflammation is associated with a reduced synthesis in like insulin-like growth factor 1 and an increased nuclear factor-κB. In stable conditions, these molecules are essential for muscle integrity. In addition, impaired uptake of long branched-chain amino acids is impaired due to endothelial reactivity and muscle perfusion caused by inflammation [27]. Furthermore, the ubiquitin-proteasome system, a mechanism of muscle fiber degradation, activates its catabolic signals in the presence of inflammation [28]. Although the evidence has been increasing recently, there is a need for more studies to identify the mechanisms by which inflammation can lead to the loss of muscle strength and mass, and subsequent disability. This is especially true for inflammatory markers other than CRP.

In this study, we reported the results of a rural sample of community-dwelling older Mexican adults. Compared to similar studies, our individuals had worse conditions except for BMI and physical activity. Regarding age, individuals in our study were older (mean = 80.6) than samples in Calvani’s study [17] mean = 78.1 and Sousa’s study [12], whose older adults had an age range between 65 and 74. Our population also had worse SPPB scores compared with similar studies. The mean of SPPB score was 6.6 in our study, while in other studies, the mean range of SPP was between 8.4 and 17.2 [12, 13, 17]. The older adults in our sample had lower BMI (mean = 24.7) compared with Calvani’s study [17] mean = 27.4 and Legrand’s study (mean BMI: 26.9 for men and 27.7 for women) [13]. In addition, our sample had better physical activity levels (58% was moderate or high) than the sample in Sousa’s study, which had 28% [12].

The results of our study should be interpreted considering the following limitations: (i) since this is a cross-sectional analysis, conclusions about causality are precluded; prospective studies are therefore required for understand association between inflammatory marker and PP. (ii) the number of selected biomarkers was small, further studies with more biomarkers are needed to understand the mechanisms of inflammation markers in older people; (iii) Life expectancy in Mexico was 75 in 2019, while the mean age in our study was 80.6, this could have an impact on the estimation of the association between CRP and low physical function because the older adults that survived have better health conditions than their counterparts who died. Despite the above limitations, this study also has some strengths. First, and as far as we know, this is one of the few studies with older adults from Latin America and the first study in Mexico to evaluate the relationship between PP and IL-10, TNF-α and CRP. Second, the physical performance was evaluated according to international recommendations of Guralnik et al. [2], which allows comparability with other studies.

## Conclusion

Our findings highlight the important role of inflammation concerning poor physical performance in older adults. Even so, the results were mixed, with a significant association of physical performance with higher CRP levels but nonsignificant with IL-10 and TNF-α. Further longitudinal research is required to incorporate a broader set of inflammatory markers to understand better the causal pathways involved in this association.

## Data Availability

The datasets used and/or analyzed during the current study are available from the corresponding author upon reasonable request.

## Abbreviations

ADL: Activities of daily living
APP: Acute-phase proteins
BMI: Body mass index
CRP: C-reactive protein
IADL: Instrumental activities of daily living
IL-10: Interleukin-10
IPAQ: International physical activity questionnaire
MET: Metabolic equivalent of task
OR: Odds ratio
PA: Physical activity
PP: Physical performance
RFS: Rural frailty study
SPPB: Short physical performance battery
TNF-α: Tumor necrosis factor-alpha
